# A tool to assess the mock community samples in 16S rRNA gene-based microbiota profiling studies

**DOI:** 10.20517/mrr.2022.18

**Published:** 2023-04-27

**Authors:** Sudarshan A. Shetty, Jolanda Kool, Susana Fuentes

**Affiliations:** ^1^Center for Infectious Disease Control, National Institute for Public Health and the Environment, Antonie van Leeuwenhoeklaan 9, Bilthoven 3721 MA, Netherlands.; ^2^Department of Medical Microbiology and Infection Prevention, Virology and Immunology Research Group, University Medical Center Groningen, Hanzeplein 1, Groningen 9713 GZ, Netherlands.

**Keywords:** Mock community, microbiome profiling, positive control

## Abstract

Inclusion and investigation of technical controls in microbiome sequencing studies is important for understanding technical biases and errors. Here, we present *chkMocks*, a general R-based tool that allows researchers to compare the composition of mock communities that are processed along with samples to their theoretical composition. A visual comparison between experimental and theoretical community composition and their correlation is provided for researchers to assess the quality of their sample processing workflows.

## INTRODUCTION

Microbiota profiling of diverse environments is widely done using 16S rRNA gene sequencing. Preparation of samples for microbiota profiling consists of sampling, storage, DNA extraction, PCR, library preparation, sequencing, and downstream bioinformatics analysis^[1-4]^. At every step, technical variability is a major factor that can ultimately affect the observed microbiota profiles^[5-8]^. Including negative and positive controls, especially mock communities with known microbial composition, is suggested to help identify technical variability and improve protocols if required^[9]^. Mock communities with known composition can be included at the step of DNA extraction (mixture of different cells) or at the PCR step (mixture of DNA from different cells). This allows for evaluating where technical variation is introduced. For example, it is known that DNA extraction methods can differently bias certain cell types, e.g., Gram-positive and Gram-negative bacteria, and that primer choice at the PCR step can neglect or favor some organisms^[5,8]^. In addition, these mock communities allow for identifying potential reagent contamination, well-to-well contamination, and to some extent, cross-sample contamination^[10-13]^. Therefore, every microbiota profiling study should include both positive and negative controls during sample processing.

Analyzing the mock community profiles and comparing them to the theoretical composition is, however, not straightforward, especially for novice microbiome scientists. A very limited number of tools are available for analyzing and comparing mock communities. The QIIME2 consists of a plugin called q2-quality-control^[14,15]^. The ZymoBIOMICS research team provides a tool called FIGARO for ZymoBIOMICS^TM^ Microbial Community Standard^[16]^. Here, we present an R-based tool, *chkMocks*, specifically designed for outputs from the R-based dada2 pipeline. The *chkMocks* R package provides a slightly different approach for investigating mock communities (see below). This tool provides support for ZymoBIOMICS^TM^ Microbial Community Standard and offers the ability to use it for custom mock communities.

## IMPLEMENTATION AND FEATURES

**Figure 1:**
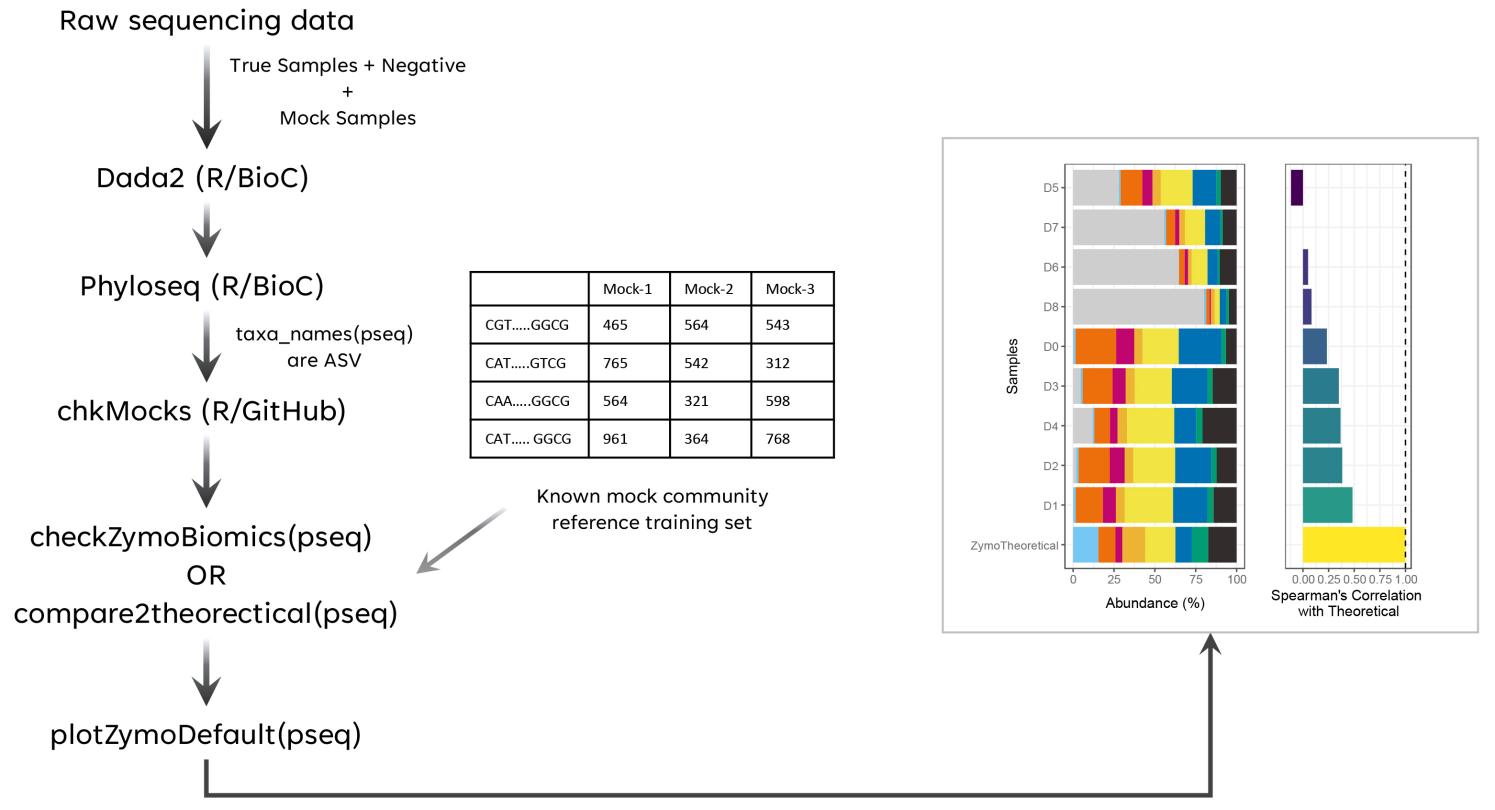
Overview of the workflow for comparing experimental mock samples with the theoretically expected composition.

**Figure 2:**
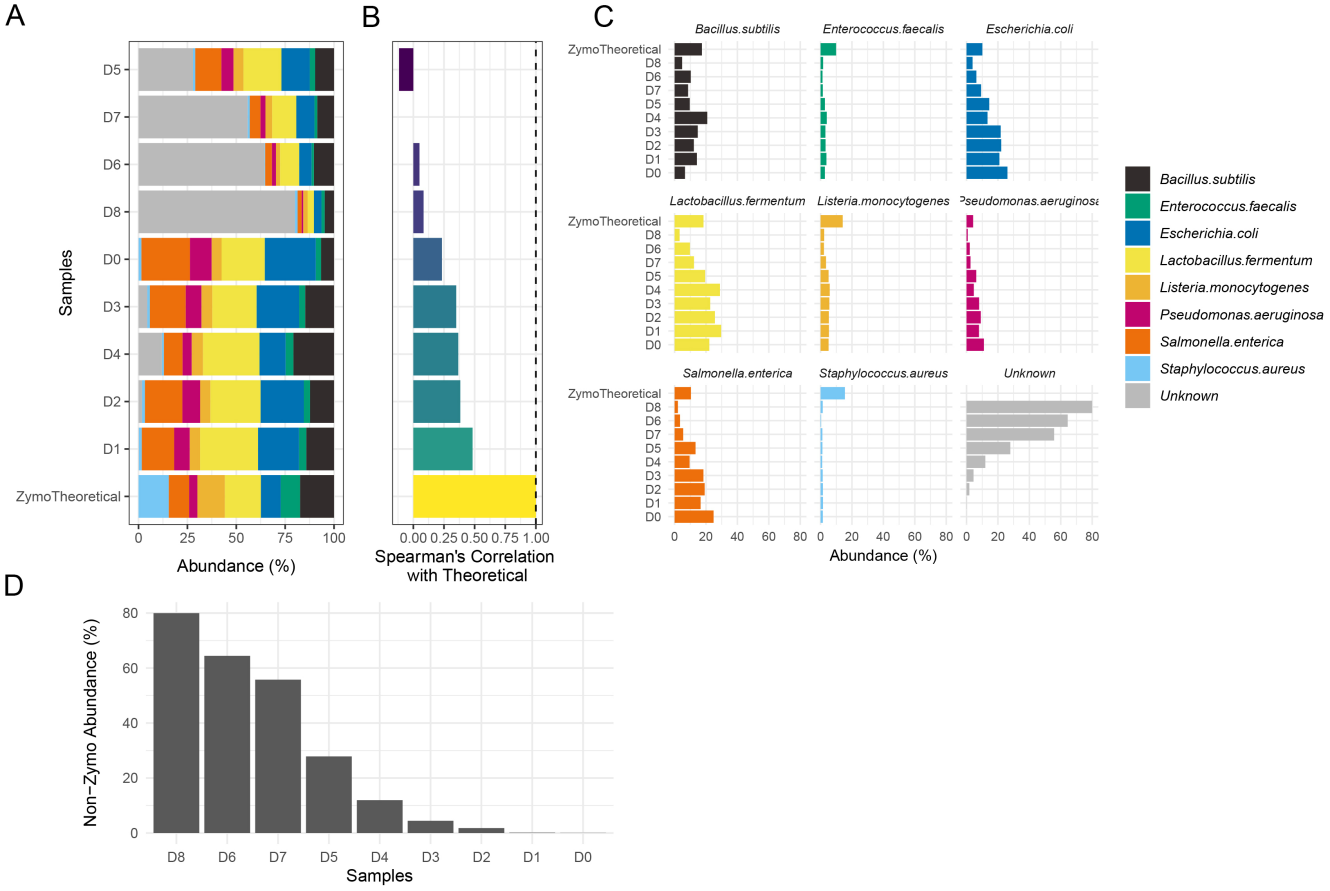
Overview of the key results generated by *chkMocks*. (A) Community composition of positive controls and expected composition of ZymoBIOMICS^TM^ Microbial Community Standard; (B) spearman’s correlation (rho) values of positive controls compared to theoretical composition; (C) percent abundances of individual taxa; (D) percent abundances of “unknown” taxa, i.e., not matching any of the standard expected taxa.

The *chkMocks* tool is implemented in R and depends on the following R packages/tools: *dada2*, *DECIPHER*, *tidyverse* tools, *microbiome*, *phyloseq* and *patchwork*^[17-22]^. An overview of the workflow/steps is depicted in Figure 1. The *chkMocks* tool requires data that completed the *dada2* workflow, from raw reads to obtaining the taxonomy assigned *phyloseq* object. The *phyloseq* object should have sequences of variants as taxa names and not be converted to text ID’s like ASV:1, etc. The *chkMocks* tool can be used by two different approaches, distinguished by the type of mock sample that is used. If users have sequenced the ZymoBIOMICS™ Microbial Community Standard (Catalog No. D6300), they can use the default *checkZymoBiomics*. For this, we have created a taxonomic training set using the FASTA files for full-length 16S rRNA gene sequences of expected microbes provided by ZymoBiomics. To demonstrate the *chkMocks* utility, we used data from a study investigating reagent contamination using the ZymoBIOMICS™ Microbial Community Standard^[10]^. Here, the Microbial Community Standard was subjected to 8 series of a 3-fold dilution (D0 to D8) and processed for 16S rRNA gene-based microbiota profiling. The outputs of *checkZymoBiomics* are (a) A *phyloseq* object with input ASVs, their abundances and taxonomic assignments; (b) A *phyloseq* object with input ASVs aggregated to species level and their abundances; and (c) A correlation table with Spearman’s correlation (rho) values of positive controls compared to theoretical composition. The user can simply plot the results with *plotZymoDefault*; this function visualizes the composition of positive controls and theoretical composition as a stacked bar plot [Figure 2A]. This is accompanied by a bar plot of Spearman’s correlation (rho) between positive controls and theoretical composition [Figure 2B]. The user can also compare the abundances of individual taxa for a clearer understanding of biases towards specific taxa [Figure 2C and D]. Here, the percentage of ‘unknown’ taxa, i.e., not matching any of the expected taxa included in the mock community, increases as dilution increases and is in agreement with values reported by the original study. All these plots provide first-hand insights to the user about the quality of their sample processing by directly comparing positive controls with expected observations.

For researchers using a custom mock community or mock communities from a different vendor, we provide a step-by-step guide on preparing the training set as a FASTA file for full-length 16S rRNA gene sequences of expected microbes using the *DECIPHER* R/BioC package. To this end, the taxonomic assignment can be done using the *assignTaxonomyCustomMock.* We provide this tutorial on the package website (https://microsud.github.io/chkMocks/) and include an example of how to compare the custom mocks with their theoretical composition. Of note, we rely on the *DECIPHER:IdTaxa* function for taxonomic assignments and *chkMocks* only supports bacteria and archaea^[23]^.

To demonstrate the application for custom mock communities, we used data from a study investigating an ASV profiling tool, NG-Tax^[24]^ and experimental samples from a previous synthetic microbiome study^[25]^. Additionally, we also provide training sets for the ZymoBIOMICS^TM^ Microbial Community Standard (Catalog No. D6331) which consists of 19 of the 21 microbes. The two fungi, *Candida albicans* and *Saccharomyces cerevisiae*, are excluded from this training set.

## CONCLUSION

The *chkMocks* was developed for the comparison of experimental mock communities with their expected compositions. The wet-lab protocols are often standardized depending on the target ecosystem that is investigated. Standardization requires analysis of positive controls, which are often microbial communities of known composition. Furthermore, a comparison of mock communities between batches when processing a large number of samples can help identify any technical variability. We developed a simple-to-use R package to ease the process of standardization and general quality check.

## DECLARATIONS

### Availability of data and materials

The *chkMocks* is implemented in the R statistical language and is released under the MIT license. The source code and associated example data are available at: https://github.com/microsud/chkMocks/.

### Copyright

© The Author(s) 2023.
